# Effects of Lanthanum Substitution and Annealing on Structural, Morphologic, and Photocatalytic Properties of Nickel Ferrite

**DOI:** 10.3390/nano13243096

**Published:** 2023-12-07

**Authors:** Thomas Dippong, Dana Toloman, Mihaela Diana Lazar, Ioan Petean

**Affiliations:** 1 Faculty of Science, Technical University of Cluj-Napoca, 76 Victoriei Street, 430122 Baia Mare, Romania; 2 National Institute for Research and Development of Isotopic and Molecular Technologies, 67-103 Donath Street, 400293 Cluj-Napoca, Romania; dana.toloman@itim-cj.ro (D.T.); diana.lazar@itim-cj.ro (M.D.L.); 3 Faculty of Chemistry and Chemical Engineering, Babes-Bolyai University, 11 Arany Janos Street, 400028 Cluj-Napoca, Romania; ioan.petean@ubbcluj.ro

**Keywords:** nickel–lanthanum ferrite, crystalline phase, specific surface, sonophotocatalysis

## Abstract

Nanoparticles of NiLa_x_Fe_2−x_O_4_ ferrite spinel incorporated in a SiO_2_ matrix were synthesized via a sol-gel method, followed by annealing at 200, 500, and 800 °C. The resulting materials were characterized via XRD, AFM, and BET techniques and evaluated for photocatalytic activity. The XRD diffractograms validate the formation of a single-phase cubic spinel structure at all temperatures, without any evidence of secondary peaks. The size of crystallites exhibited a decrease from 37 to 26 nm with the substitution of Fe^3+^ with La^3+^ ions. The lattice parameters and crystallite sizes were found to increase with the rise in La^3+^ content and annealing temperature. Isotherms were employed to calculate the rate constants for the decomposition of malonate precursors to ferrites and the activation energy for each ferrite. All nanocomposites have pores within the mesoporous range, with a narrow dispersion of pore sizes. The impact of La content on sonophotocatalytic activity was evaluated by studying Rhodamine B degradation under visible light irradiation. The results indicate that the introduction of La enhances nanocomposite performance. The prepared Ni-La ferrites may have potential application for water decontamination.

## 1. Introduction

Nickel ferrite (NiFe_2_O_4_) stands out as one of the most prominent in the spinel ferrite class. In bulk, it exhibits a rhombohedrally distorted cubic structure, characterized by an inverse spinel structure; this arrangement involves an antiparallel spin alignment between Fe^3+^ and Ni^2+^ ions on octahedral sites, while equal Fe^3+^ ions are positioned at the tetrahedral sites [1,2,3,4]. The unit cell of NiFe_2_O_4_ is composed of 32 O^2−^, 16 Fe^3+^, and 8 Ni^2+^ ions. The oxygen ions form 32 octahedral sites (B-sites) and 64 tetrahedral ones (A-sites). These sites have the capacity to host a total of 24 cations. Within this inverse spinel structure, an exclusive occupation of A-sites by Fe^3+^ ions arises, whereas the B-site is shared by Ni^2+^ and Fe^3+^ ions, both demonstrating electron exchange at the octahedral site, highlighting their unique electrical and magnetic properties [5]. The superior characteristics of nickel ferrites, such as a low permittivity superparamagnetism and favorable optical band gap (Eg) values, make them suitable for high-frequency applications [2,4,5,6]. The incorporation of La^3+^ ions into spinel ferrites induces a strong spin–orbit coupling of their angular momentum, resulting in enhanced dielectric properties [7]. The presence of the rare earth ion in the spinel ferrite contributes to improved densification, electrical resistivity, and reduced eddy current losses [7]. Their unique properties make them suitable for a wide range of applications such as photoacoustic imaging, transformer cores, biosensors, high-density storage media, electron transport devices, hyperthermia, analog devices, imaging, biological field, radio frequency, microwave absorbing materials, water treatment, gas sensors, lithium-ion batteries, spin canting, surface anisotropy, and superparamagnetism [1,2,3,4,6].

The properties of Ni-La ferrite nanoparticles are intricately linked to the chosen synthesis method, which impacts both composition and microstructure [8]. Several synthesis methods are worth mentioning, including ball milling, sol–gel, solid-state reaction, spray pyrolysis, microemulsion, thermal pyrolysis, hydrothermal, solvothermal, citrate gel auto-combustion, self-propagating high-temperature synthesis, microwave, or chemical coprecipitation [1,2,3,4,5,6,7,8,9]. Sol–gel is arguably the most versatile method, as it requires less time and offers a reduced cost due to the low temperature requirement; it is highly reproducible, allowing for a good stoichiometry control that leads to a homogenous, single-phase final product under normal ambient conditions [1,2,3,4,5,6]. It is worth mentioning that the annealing process influences both phases and the increase in crystallite size [10]. The incorporation of NiLa_x_Fe_2−x_O_4_ into a mesoporous SiO_2_ matrix plays a crucial role in enhancing water stability, improving biocompatibility, and mitigating the degradation of NiLa_x_Fe_2−x_O_4_ nanoparticles [11,12,13,14]. The SiO_2_ coating not only prevents agglomeration by regulating dipolar attraction between nanoparticles but also facilitates the binding of biomolecules on the mesoporous SiO_2_ surface, enabling targeted ligands and drug loading on the nanocarrier surface [11,12,13,14]. The synthesis of NiLa_x_Fe_2−x_O_4_ embedded in the SiO_2_ matrix following a sol–gel method involves the mixing of reactants with tetraethyl orthosilicate (TEOS), forming strong networks with moderate reactivity that allow the incorporation of various inorganic and organic molecules [11,12,13,14]. Simple adjustments in synthesis conditions, such as pH, time, and annealing temperature, can provide more precise control over nucleation and particle growth [11,12,13,14].

Photocatalytic properties are highly dependent upon parameters such as surface area, particle size, and concentration of dopants [15]. The photochemical process occurs at the surface of metal oxides, involving two types of reactions, namely oxidation (resulting in positive holes) and reduction (producing negative electrons) [4]. By tuning the band gap energy (Eg) of ferrites below 3 eV, one can improve upon their photocatalytic properties [6]. In spite of its oxidation capacity, the use of a wide UV band gap, and noteworthy photocatalytic activity, to achieve better efficiency proves to be a challenge due to rapid recombination. Due to their use of visible light, ferrites with lower Eg values are suitable for applications like wastewater treatment in pollutant degradation [6]. According to Zhang et al. [16], conventional homogeneous photocatalysis is characterized by inherent drawbacks, including the easy recombination of photo-induced electron–hole (e^−^/h^+^) pairs and light absorption restricted to the ultraviolet region. This study proposes that the development of heterogeneous photocatalysis has proven to be an effective strategy for expanding the range of light absorption wavelengths and enhancing the separation of charge carriers [16]. Another study, by Shah et al. [17], highlights the inclusion of a new energy level in between the conduction and valence bands of TiO_2_ and NiFe_2_O_4_, thus facilitating the separation of photoinduced electrons and holes. The investigation of Zhang et al. [16] emphasizes the stability of a TiO_2_/Ag/SnO_2_ photocatalyst following a methylene blue degradation over four cycles. Ghoneim [18] evaluates the potential of using Cu_0.3_Cd_0.7_CrFeO_4_ nano-spinel for cost-effective wastewater treatment. Padmapriya et al. [19] note that the zinc–ferrite nanoparticle photocatalysis depends on surface area and particle size. Additionally, sonocatalysis, utilizing ultrasound for pollutant degradation, combines effectively with photocatalysis in the versatile sonophotocatalysis technique [20].

This study explores the synthesis, structural aspects (crystallite size and lattice parameter), surface characteristics (specific surface area and porosity), morphology (particle size, roughness, and height), and the sonophotocatalytic performance of Ni-La ferrites incorporated into SiO_2_. These nanocomposites were prepared using a sol–gel method followed by thermal treatment. The crystalline phases, crystallite sizes, and lattice constants were examined via X-ray diffraction (XRD). The Ni-La-Fe ferrite composition was investigated via inductively coupled plasma optical emission spectrometry (ICP-OES). Specific surface area (SSA) and porosity were determined by analyzing N_2_ adsorption–desorption isotherms. Particle attributes, including shape, size, size distribution, and agglomeration, were characterized using atomic force microscopy (AFM). The sonophotocatalytic degradation of the samples was evaluated under visible light exposure with concurrent sonication using a Rhodamine (RhB) solution.

## 2. Materials and Methods

Ni(NO_3_)_2_∙6H_2_O, La(NO_3_)_2_∙6H_2_O, Fe(NO_3_)_3_∙9H_2_O, 1,3-propandiol (1,3PD), TEOS, and ethanol were used to synthesize Ni-La ferrites embedded in a SiO_2_ matrix (50% wt. ferrite, 50% wt. SiO_2_) using a sol–gel method. Ni:La:Fe molar ratios of 10:1:19 (NiLa_0.1_Fe_1.9_O_4_/SiO_2_), 10:3:17 (NiLa_0.3_Fe_1.7_O_4_/SiO_2_), 10:5:15 (NiLa_0.5_Fe_1.5_O_4_/SiO_2_), 10:7:13 (NiLa_0.7_Fe_1.3_O_4_/SiO_2_), 10:9:11 (NiLa_0.9_Fe_1.1_O_4_@SiO_2_), 10:11:9 (NiLa_1.1_Fe_0.9_O_4_@SiO_2_), and a nitrate:1.3PD:TEOS molar ratio of 1:1:1 were used. The as-produced sols were maintained at room temperature for gelation and the process was followed by grinding and drying at 200 °C (6 h), heating at 500 °C (6 h), and annealing at 800 °C (6 h).

The structural characterization was explored via X-ray diffraction using a Bruker D8 Advance diffractometer in normal temperature conditions (with CuKα radiation, λ = 1.5406 Å), running at 40 kV and 40 mA. The content of Ni, La, and Fe in the ferrites was confirmed through an inductively coupled plasma optical emission spectrometry (ICP-OES) using an Optima 5300 DV (Perkin Elmer, Waltham, MA, USA) spectrometer, after an aqua regia microwave digestion employing a Berghof Speedwave Xpert system. atomic force microscopy (AFM) was effectuated in AC mode with a JEOL Scanning Probe Microscope 4210 (Jeol Company, Akashima, Japan) using sharp probes (NSC 15 produced by Mikromasch Company, Watsonville, CA, USA) featuring a resonance frequency of 325 kHz and a spring constant of 40 N/m. The annealed powders were dispersed in deionized water to release nanoparticles which were adsorbed on the solid substrate (e.g., glass) as thin layers. Three different areas of 1 μm^2^ were scanned for each specimen. The topographic characteristics were measured using JEOL WinSPM 2.0 processing software (Jeol Company, Akashima, Japan). The shape and clustering of the particles were examined by depositing dried sample suspensions onto a copper grid coated with a thin carbon film using a Hitachi HD-2700 transmission electron microscope (Hitachi, Tokyo, Japan). Specific surface area (SSA) and porosity parameters (mean pore size and pore size distribution) were calculated from N_2_ adsorption–desorption isotherms using the BET method (for SSA) and the Dollimore–Heal model (for porosity). The isotherms were recorded on a Sorptomatic 1990 instrument (Thermo Fisher Scientific, Waltham, MA, USA). A V570 model UV-VIS-NIR spectrophotometer (JASCO, Oklahoma City, OK, USA) containing the absolute reflectivity accessory (ARN-475, JASCO) was used to register the UV–VIS absorption spectra. The optical band gap was determined from Tauc’s relationship. The sonophotocatalytic efficacy was assessed using a Rhodamine (RhB) solution exposed to visible light within a Laboratory-Visible-Reactor system, using a 400 W halogen lamp (Osram, Munich, Germany) and an ultrasonic bath. In this experimental setup, 10 mg of catalyst was blended with a 20 mL solution of 1.0 × 10^−5^ mol/L RhB in water, and the resulting mixture was stirred in darkness until adsorption equilibrium was achieved on the catalyst surface. Each photodegradation test spanned 240 min, with 3.5 mL samples extracted every 60 min for subsequent analysis. Following catalyst removal, the absorbance of the RhB solution was measured at 554 nm. Sonophotocatalytic activity was determined based on the degradation rate. Prior to sonophotodegradation tests, the adsorption of RhB on the nanoparticle surface was assessed. This involved mixing the photocatalyst with the RhB solution in the dark for 60 min until adsorption–desorption equilibrium was reached. The photodegradation of RhB was modeled using the first-order kinetic model, relying on the absorbance data. To demonstrate the generation of reactive oxygen species (ROS) by the samples, we employed the EPR Bruker E-500 ELEXSYS X-band spectrometer (Bruker, Billerica, MA, USA) (9.52 GHz), coupled with the spin trapping technique. The spin trapping reagent used for this purpose was 5,5-Dimethyl-1-pyrroline N-oxide (DMPO).

## 3. Results and Discussion

**Figure 1:**
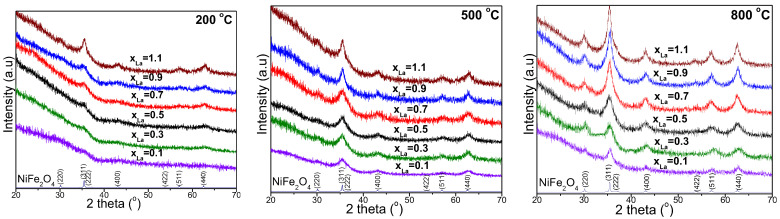
XRD patterns of NiLa_x_Fe_2−x_O_4_/SiO_2_ (x_La_ = 0.1, 0.3, 0.5, 0.7, 0.9, 1.1) annealed at 200, 500, and 800 °C.

The XRD patterns of NiLa_x_Fe_2−x_O_4_/SiO_2_ (x = 0.1–1.1) nanocomposites annealed at 200, 500, and 800 °C are presented in Figure 1. At 200 °C in case of low La content, the formation of the weakly crystalline Ni-La ferrite phase can be observed (NiFe_2_O_4_ (JCPDS card no 89-4927) and La_0.14_Fe_3_O_4_ (JCPDS card no. 75-8137)). The NiFe_2_O_4_ (x = 0.1) crystalline phase originates from a combination of Ni’s low oxidation capacity, low melting point, high electronegativity, high thermal expansion coefficient, and high specific heat capacity [1]. At 200 °C, the broad peak observed at 2θ =20–30° suggests a low level of crystallization in the nanocomposites. At 500 and 800 °C, the intensity of the diffraction peaks increases due to a better crystallization of ferrites. The existence of reflection planes such as (220), (311), (222), (400), (331), (422), (511), and (440) confirms the distinct phase of Ni-La ferrite, characterized by a face-centered cubic inverse spinel structure in the *Fd-3m* space group [1]. All the samples revealed a homogeneous phase spinel Ni-La ferrite without any impurities being registered in the XRD patterns. The peak intensity of the (311) peak decreased with the increase in La content and increased with the increase in thermal treatment temperature. The diffraction peaks sharpen, and their intensity increases with rising annealing temperatures, which are attributed to pronounced agglomeration without immediate recrystallization; this process leads to the formation of a single crystal rather than a polycrystal [21].

The average crystallite size (D_C_) increases from 19.5 to 35.4 nm at 800 °C, with an increase in the Fe^3+^ substitution by La^3+^ ion (Table 1). The increase in D_C_ with temperature is attributed to the agglomeration of crystallites without recrystallization, resulting in a transition from a polycrystalline structure to single crystals at elevated temperatures [11,12,13,14]. Another plausible explanation is the coalescence process, with small nanoparticles merging into larger ones at high annealing temperatures [1,11,12,13,14]. The substitution of La^3+^ for Fe^3+^ induces crystalline anisotropy following the substantial difference in their sizes, with the doping of La^3+^ ions acting as a kinetic barrier to further grain growth [7,22]. Increasing the temperature distinctly enhances the crystallinity of lanthanum–nickel ferrite [10]. The amorphous phase dominates at low annealing temperatures and undergoes partial transformation into various crystalline phases at higher annealing temperatures [1]. The increase in D_C_ indicates a reduction in the densities of nucleation centers [1]. La–O bond energy is greater when compared with that of Fe–O; as such, the replacement of Fe^3+^ ions with La^3+^ ions at the octahedral site in NiFe_2_O_4_ causes La^3+^ ions to enter the interstitial location and hinders nickel ferrites from crystallizing [6]. The variation in D_C_ may also be attributed to the peak broadening associated with lattice strains, the grain growth blocking effect induced by the SiO_2_ matrix, as well as thermal and instrumental effects [1,11,12,13,14]. Following Vegard’s law, the increase in lattice parameter (a) with the increase in lanthanum concentration could be explained on the basis of ionic radii of the La^3+^ ion substituted in the structure [1,2,3,9,22]. In nickel ferrite, Ni^2+^ ions predominantly occupy octahedral sites (B-site), whereas Fe^3+^ ions occupy both tetrahedral (A) and octahedral (B) sites. The increase in the lattice constant (a) following the increase in La content (Table 1) can be attributed to the larger ionic radius of La^3+^ ions (oct: 1.06 Å) when compared with that of Fe^3+^ ions (oct: 0.67 Å); the La^3+^ ions of higher radii substitute Fe^3+^ ions of smaller radii at the octahedral sites [1,2,3,6,9,22]. The inverse spinel structures cause a partial migration of Ni^2+^ ions from A to B sites; the migration is consorted by an opposite relegation of the corresponding numerical values of Fe^3+^ ions from B to A sites in order to relax the strain [1,2,3,11,12,13,14].

Based on the content of Ni, La, and Fe within the samples, the Ni/La/Fe molar ratio was calculated and compared with the theoretic value for each sample (Table 2). A good agreement was observed between the theoretical and experimental molar ratios across all samples and calcination temperatures.

**Figure 2:**
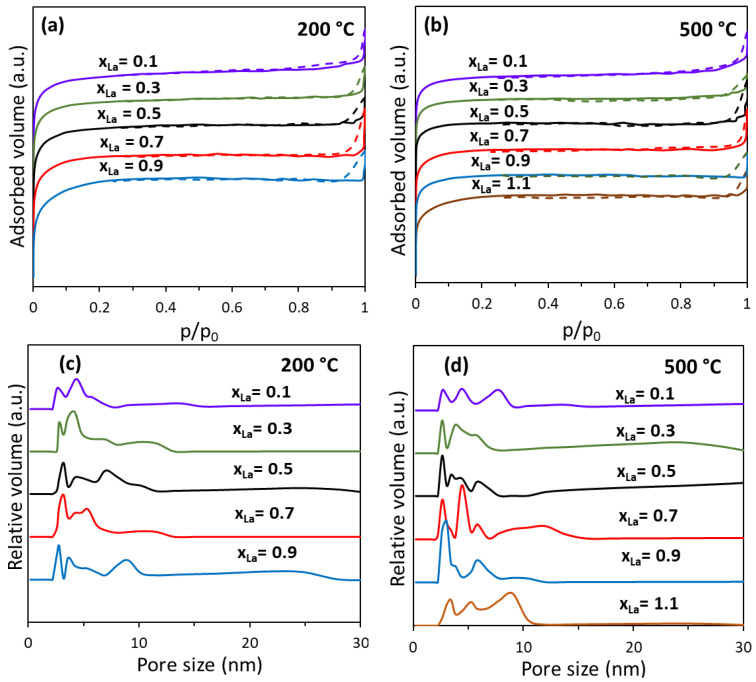
Nitrogen adsorption–desorption isotherms (**a**,**b**) and pore diameter distribution (**c**,**d**) for NiLa_x_Fe_2−x_O_4_/SiO_2_ (x_La_ = 0.1, 0.3, 0.5, 0.7, 0.9, 1.1) annealed at 200 °C and 500 °C. (full line—adsorption and dashed line—desorption branch of the isotherms).

The nitrogen adsorption–desorption isotherms recorded at −196 °C are utilized to provide information about the porous structure and the surface area of the nanocomposites. The isotherm shapes observed in the composites annealed at 200 °C and 500 °C exhibit characteristics typical of mesoporous materials, all falling into the type IV category according to the IUPAC classification. Additionally, they display minimal hysteresis at high relative pressures [23]. For the materials thermally treated at higher temperatures (800 °C), the isotherms could only be recorded for the nanocomposite samples with lower lanthanum content: x_La_ between 0.1 and 0.5. For the composites with higher La concentration, the surface area is below the detection limit of the equipment (below 0.5 m^2^/g). As can be observed in Figure 2, the shape of the isotherms is comparable for all nanocomposites annealed at 200 °C and 500 °C, suggesting a very similar porous structure of these materials. For the nanocomposite samples calcined at the same temperature, the SSA values vary only in moderate proportion to the lanthanum concentration. A direct, linear correlation between the lanthanum content and the specific surface area was not observed (Table 1). For the samples annealed at 200 °C, there is a general trend of a slow decrease in the SSA with the increase in La substitution within the ferrite lattice. However, no such trend can be observed for the samples calcined at 500 °C. In this case, after an initial decrease in SSA with the increase in La content, the trend reverses and the SSA started to increase again, with the sample with the lowest value for SSA being NiLa_0.5_Fe_1.5_O_4_/SiO_2_ (173 m^2^/g compared with 230 m^2^/g for NiLa_0.1_Fe_1.9_O_4_/SiO_2_ and 240 m^2^/g for NiLa_1.1_Fe_0.9_O_4_/SiO_2_, respectively).

This trend corresponds to the one observed for surface roughness (Rq) of the film prepared for AFM analysis. For the samples containing the same amount of La but thermally treated at different temperatures, the increase in temperature led to a decrease in SSA. This behavior was previously reported for nickel ferrite [24], ferrite-SiO_2_ composite materials [25], as well as other oxides [26], being usually related to an increase in crystallinity due to crystallite growth (Table 1 and Figure 1) and/or to the prevalence of silica crystalline forms with lower surface areas. All the samples annealed at 800 °C present very low surface areas (less than 3 m^2^/g) indicating that in the samples calcined at this temperature, the porous structure is no longer present. For the samples calcined at 200 °C and 500 °C, the distribution in pore sizes (Figure 2c,d) confirms the mesoporous structure of NiLa_x_Fe_2−x_O_4_/SiO_2_. The pore dimensions for all samples are low, with values less than 10 nm, and are thus situated in the lower region of the mesoporous domain. All the tested nanocomposites present a multimodal pore size distribution that is very similar across all samples. The size distribution is relatively narrow (3–10 nm), with the multiple pore dimensions inside this range being characteristic of composite materials in which the global porous structure is given by the combined effect of the porosity of each component

**Figure 3:**
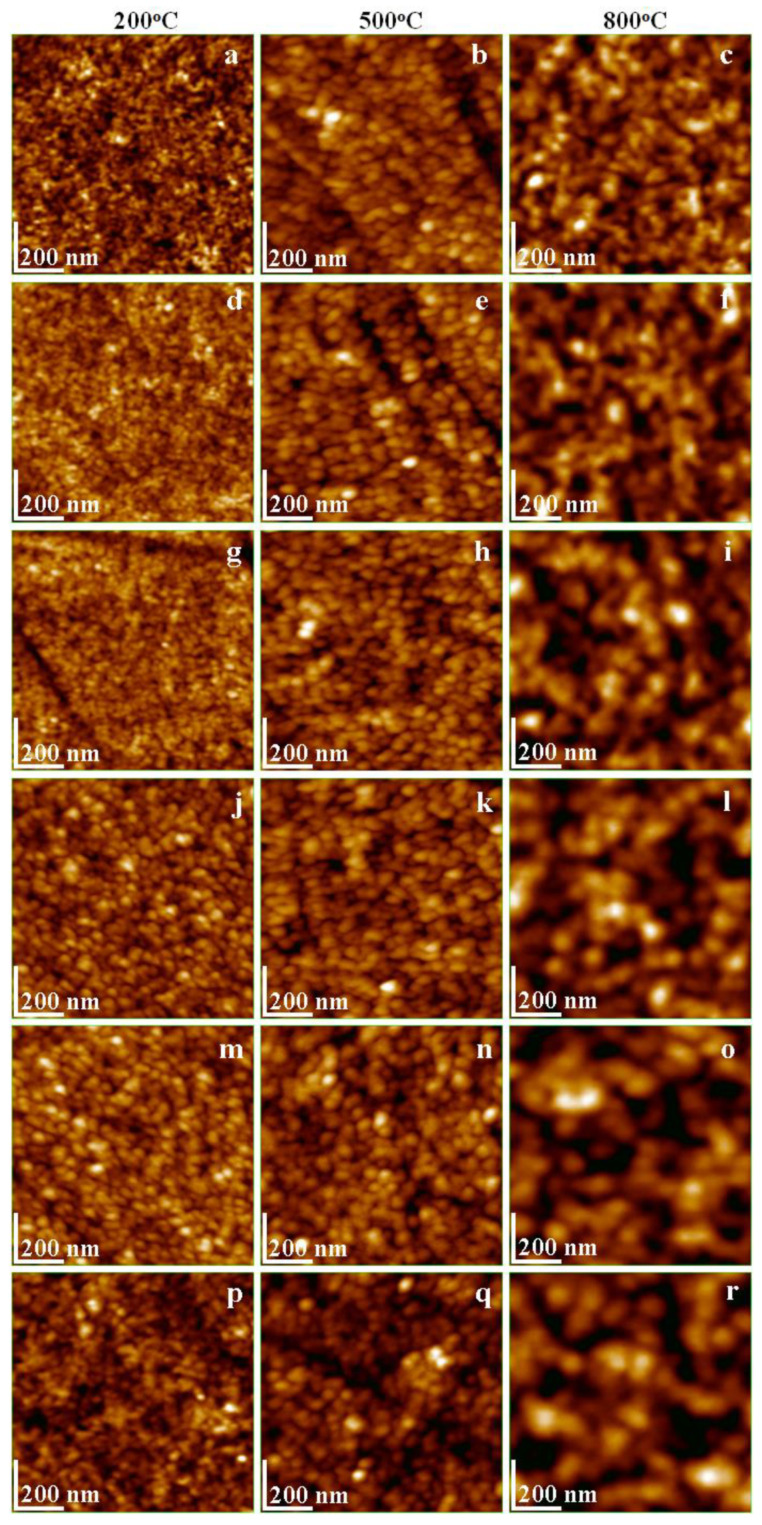
AFM topographical images of NiLa_x_Fe_2−x_O_4_/SiO_2_ annealed at 200, 500, and 800 °C. (**a**–**c**) x_La_ = 0.1; (**d**–**f**) x_La_ = 0.3; (**g**–**i**) x_La_ = 0.5; (**j**–**l**) x_La_ = 0.7; (**m**–**o**) x_La_ = 0.9; (**p**–**r**) x_La_ = 1.1.

The ferrite powder obtained after annealing is slightly agglomerated. Therefore, each sample was dispersed into deionized water and transferred onto glass substrates via vertical adsorption. The intense Brownian movement within dispersed particles promotes their individualization, with these nanoparticles being attracted to the glass surface and subsequently adsorbing, forming a thin film [27,28]. The obtained ferrite thin films were investigated using AFM microscopy and the obtained topographic images are presented in Figure 3. A small substitution of Fe atoms with La (x_La_ = 0.1) within nickel ferrites has limited influence on particle size and shape and is mainly observed only at the crystalline lattice level as revealed by XRD patterns in Figure 1. Thus, the AFM image of the sample treated at 200 °C reveals very small rounded particles of about 10 nm in diameter, as shown in Figure 3a. As no crystallites were observed in the XRD analysis results, we assume that these nanoparticles are amorphous. Increasing the annealing temperature to 500 °C, a diameter enhancement to 22 nm was observed, as shown in Figure 3b. This observation is in good agreement with ferrite crystallite development. The ferrite core has a crystallite of 10.4 nm, which is covered with amorphous silica up to the observed diameter of nanoparticles. 

The crystallization process is enhanced at 800 °C, developing a ferrite core of 19.5 nm that conducts to the formation of larger nanoparticles of 33 nm in diameter, as shown in Figure 3c. The crystalline core introduces certain sharp corners to the shape of the nanoparticles, but these corners are rounded by the amorphous silica glaze. This behavior aligns with AFM observations made on nickel ferrite [29,30]. Increasing the amount of La substitution with x_La_ = 0.3, less significant changes in the obtained nanoparticles after annealing at 200 °C and 500 °C were found. The exception here is a slight increase in their diameters, as shown in Figure 3d,e. The major change occurs after annealing at 800 °C, as shown in Figure 3f, where the nanoparticles have an increased size of about 38 nm with a ferrite crystalline core of 22.8 nm (as calculated from XRD patterns) and their shape becomes boulder-like with rounded corners due to the cubic FCC crystals’ expansion. This tendency is progressively accentuated by increasing x_La_ from 0.5 to 0.9. Smaller rounded particles are observed after annealing at 200 °C, exhibiting a gradual increase in size from approximately 15 to 20 nm. This size evolution is attributed to the development of small ferrite crystallites and the presence of an amorphous silica coating, as shown in Figure 3g,j, and m. A similar enhancement from a diameter of about 28 nm to one of 35 nm is observed after annealing at 500 °C, with the development of a vigorous ferrite core crystallite (as determined from XRD patterns); however, it was not strong enough to alter the rounded shape of the nanoparticles, as shown in Figure 3h,k,n. 

The situation is more favorable after annealing at 800 °C because the nanoparticles are well developed, evidencing a small and constant increase in the diameter from 42 to 46 nm along with the accentuation of their boulder aspect as a consequence of cubic FCC single-phase development through the ferrite core, as shown in Figure 3i,l,o. The high amount of La substitution of Fe atoms within nickel ferrite (x_La_ = 1.1) has a major impact on the nanostructure of all particles. The shape remains rounded after the annealing at 200 °C. However, the diameter increases at 21 nm with a ferrite crystallite core of 9.8 nm, as shown in Figure 3p, and is comparable to x_La_ = 0.1 annealed at 500 °C, Figure 3b. The condition is further improved following annealing at 800 °C, resulting in nanoparticles with a diameter of approximately 37 nm.

These nanoparticles exhibit a crystalline core measuring 22.6 nm, and the initially rounded shape shows a slight alteration with the emergence of square corners, as depicted in Figure 3q. These features are not readily apparent due to the presence of the amorphous silica glaze, but they are clearly indicated by the XRD pattern. The nanoparticles exhibit significant elongation of the boulder shape, attributed to the strong development of the ferritic core following annealing at 800 °C, as illustrated in Figure 3r, resulting in a size of approximately 50 nm and a crystallite core measuring 35.4 nm. The substitution of Fe atoms with La appears to be facilitated by higher annealing temperatures, promoting the formation of topographically anisotropic nanoparticles. This distinctive assembly at the nanostructural level suggests the potential for special properties. 

**Figure 4:**
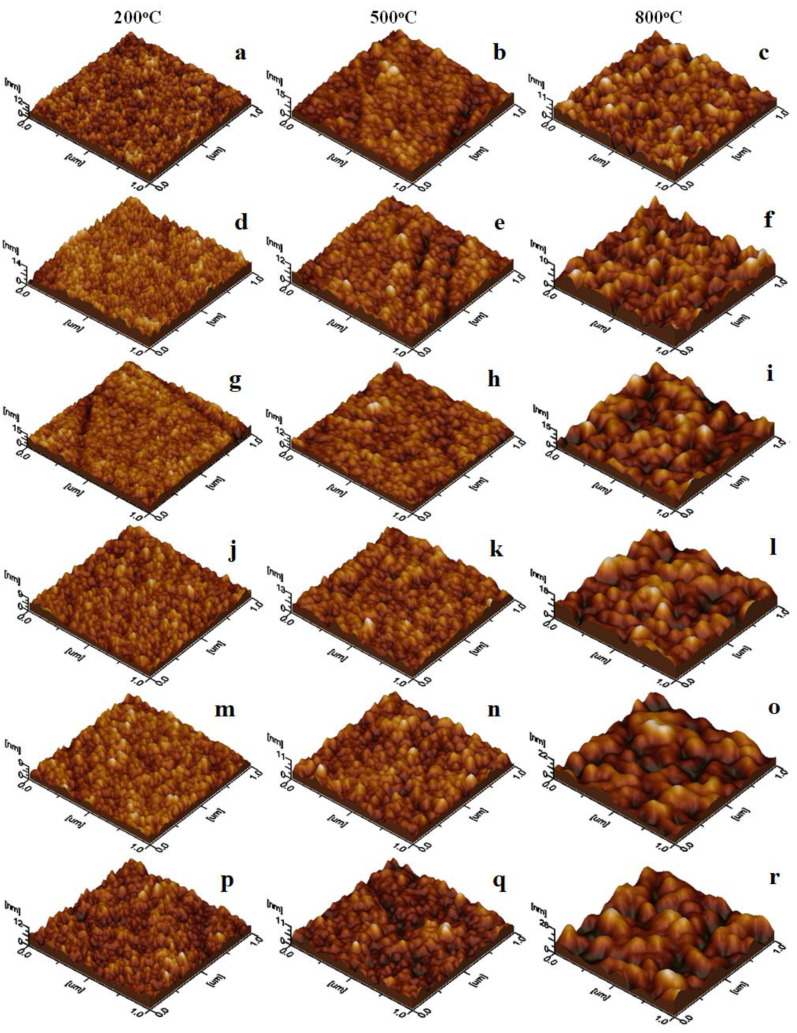
AFM tridimensional profiles of NiLa_x_Fe_2−x_O_4_/SiO_2_ annealed at 200, 500, and 800 °C. (**a**–**c**) x_La_ = 0.1; (**d**–**f**) x_La_ = 0.3; (**g**–**i**) x_La_ = 0.5; (**j–l)** x_La_ = 0.7; (**m**–**o**) x_La_ = 0.9; (**p**–**r**) x_La_ = 1.1.

By examining the three-dimensional profiles of the resulting thin films in Figure 4, one can observe that nanoparticles annealed at 200 °C produce smooth, uniform, and compact layers characterized by low heights and minimal surface roughness, as indicated in Table 1. As the annealing temperature rises, the nanoparticle diameter expands, and the thin film becomes more agglomerated, resulting in localized unevenness that increases both height and surface roughness. Notably, nanoparticles obtained after annealing at 800 °C exhibit excellent individualization, and the adsorbed thin film is less compact. This phenomenon is linked to the augmented diameter, leading to a notable increase in surface roughness. These findings hold potential for the future application of customized surfaces through thin film deposition. Achieving desired properties may involve adjusting the nanoparticle range appropriately.

**Figure 5:**
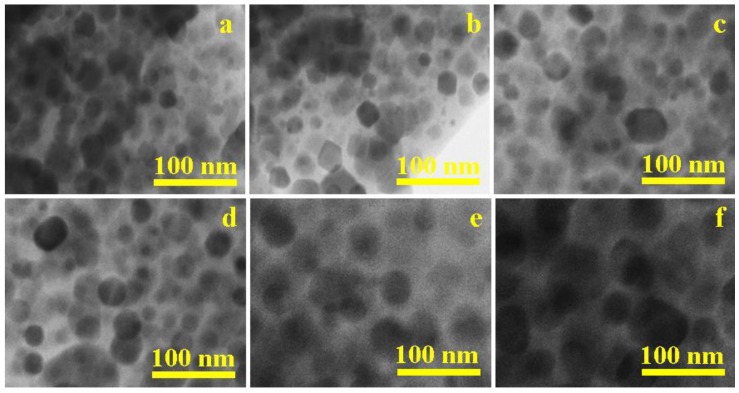
TEM images of NiLa_x_Fe_2−x_O_4_/SiO_2_ annealed at 800 °C. (**a**) x_La_ = 0.1; (**b**) x_La_ = 0.3; (**c**) x_La_ = 0.5; (**d**) x_La_ = 0.7; (**e**) x_La_ = 0.9; (**f**) x_La_ = 1.1.

The morphological characteristics of NiLa_x_Fe_2−x_O_4_/SiO_2_ samples annealed at 800 °C (x_La_ = 0.1, 0.3, 0.5, 0.7, 0.9, 1.1) were investigated using transmission electron microscopy (TEM), as illustrated in Figure 5. TEM images clearly reveal the spherical nature and uniform size distribution of particles. Also, the mean size of particles increases from 26 nm to 47 nm with rising La^3+^ content, which is likely influenced by higher surface tension in smaller nanoparticles, driving increased agglomeration [1,2,3,6,9]. Spherical particle shapes may be attributed to the synthesis method and surface properties, while agglomeration could result from interfacial surface tension phenomena [1,2,3,6,9]. Discrepancies between crystallite size (D_XRD_), particle size from AFM data (D_AFM_), and TEM-derived particle size (D_TEM_) may be explained by interference from the amorphous SiO_2_ matrix and large nanoparticles in diffraction patterns [11,12,13,14]. Agglomeration could be explained by the influence of thermal treatment temperature and potential surface defects [3]. 

**Figure 6:**
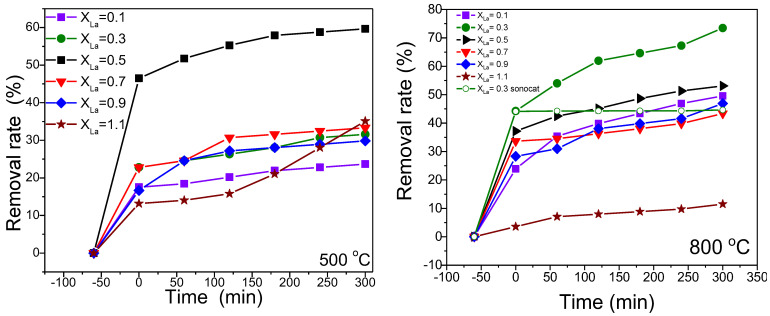
RhB solution degradation rate under visible irradiation in the presence of the 500 and 800 °C tested samples.

The sonophotocatalytic performance of the samples was examined when exposed to visible irradiation using a RhB synthetic solution. Prior to the irradiation, the samples were subjected to 60 min of darkness to achieve the adsorption equilibrium. The results of this investigation are depicted in Figure 6.

For samples subjected to annealing at 500 °C, the adsorption capacity falls within the range of 12% to 22%, except for NiLa_0.5_Fe_2.5_O_4_/SiO_2_, which exhibits a notably higher adsorption capacity at around 45%. An interesting observation is the fact that this sample has the smallest specific surface area. The adsorption capacity increases for samples annealed at 800 °C, ranging from 22% to 45%, excluding NiLa_1.1_Fe_0.9_O_4_/SiO_2_ nanocomposites with an adsorption capacity of less than 5%. As expected, all samples annealed at 800 °C, with the exception of that with x_La_ = 1.1, have lower specific surface area and still have higher adsorption capacity. Based on this observation, it can be concluded that the adsorption capacity of these samples might be attributed to interactions between the surface functional groups of the prepared ferrites and the active functional groups of RhB [31]. Figure 6 illustrates the removal rates of the samples after 5 h of irradiation. Among samples annealed at 500 °C, NiLa_0.5_Fe_1.5_O_4_/SiO_2_ demonstrates the most effective removal rate at approximately 60%. However, as the annealing temperature rises to 800 °C, the removal rate of this sample decreases to 53%, which is attributed to its larger crystallite size dimension (D_C_) and lower specific surface area (SSA). Notably, in the samples annealed at 800 °C, NiLa_0.5_Fe_1.5_O_4_/SiO_2_ is surpassed in removal rate by NiLa_0.3_Fe_1.5_O_4_/SiO_2_, reaching a maximum removal rate of 73%. This value is higher than the previously reported removal rate of Ni-ferrite of about 39% [32]. To assess the impact of ultrasound, this sample was kept in the dark for an additional 300 min, and the results are incorporated in Figure 6. It can be observed that ultrasound does not have a significant effect on the sample removal rate.

The first-order kinetic model was applied to describe the photocatalytic process (1):

(1) $$-ln\frac{A_{t}}{A_{0}^{*}}=k\times t,$$

where A_t_ represents RhB absorbance at t time; $A_{0}^{*}$ is the absorbance of RhB after dark adsorption; t—irradiation time; and k—apparent kinetic constant. 

**Figure 7:**
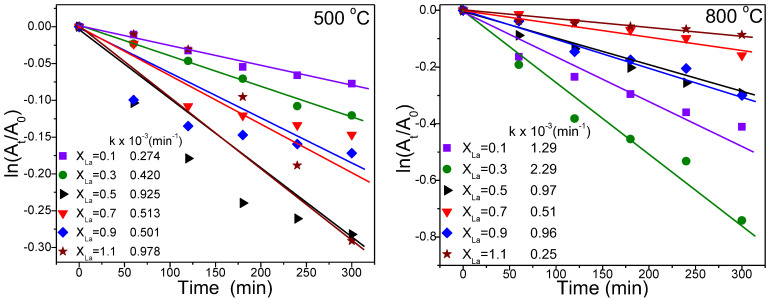
Photodegradation kinetics of RhB synthetic solution at 500 and 800 °C.

The experimental data were fitted using the rate equation, and the resulting plots, demonstrating a linear correlation with irradiation time, are showcased in Figure 7.

The inset of Figure 7 displays the rate constant values derived from the fitting process. Upon analyzing the obtained results, it is evident that the NiLa_0.3_Fe_1.7_O_4_/SiO_2_ sample, annealed at 800 °C, exhibited the most superior photocatalytic activity.

**Figure 8:**
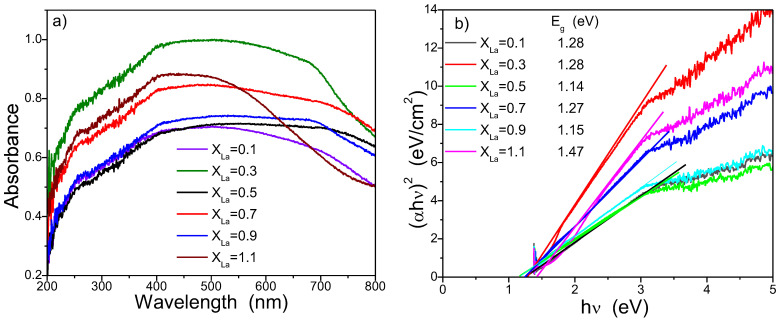
(**a**) UV-Vis absorption spectra of the samples. (**b**) Tauc’s plot.

The photocatalytic activity is influenced by various factors, and one key factor is the band gap energy. To assess this, we determined the band gap energy using Tauc’s equation based on the UV-Vis absorption spectra. In Figure 8a, the UV-Vis absorption profiles of all samples annealed at 800 °C are presented. The significant absorption is a result of electron excitation from the valence band to the conduction band. The variation in absorbance band edge is attributed to the presence of interface defects, point defects, and interactions involving photogenerated electrons [1]. The UV–visible spectrum results from electronic transitions, moving from a lower energy band to a higher energy band. In the case of nickel ferrite, this transition is attributed to electrons moving from the O 2p level to the Fe 3d level. This is explained by considering the O 2p orbital as the valence band and the Fe 3d orbital as the conduction band, as the band structure is primarily defined in this manner [33]. Through a substitution with La ions, the maximum absorption and the absorption edge vary; however, all samples exhibit a broad response across the entire visible range.

Figure 8b shows the Tauc plot of (αhv)^2^ versus hv for the direct allowed transition of all samples. The extrapolation of this plot by linear region to the point = 0 gives the corresponding values of the direct band gap. The optical band gaps were calculated and are inserted in the inset of Figure 8b. The calculated values indicate a significant decrease in the band gap energies of NiLa_0.5_Fe_1.5_O_4_/SiO_2_ and NiLa_0.9_Fe_1.1_O_4_/SiO_2_ when compared with the 1.45 eV band gap of Ni-ferrite reported in a previous study [34]. This reduction is likely attributed to the introduction of additional dopant levels into the band gap of Ni ferrite. In accordance with Rajeshwari et al. [34], the band gap values for the prepared Lanthanum-doped manganese nanoferrite range from 1.89 to 2.35 eV, showing improvement compared with the 1.25–1.38 eV band gap values of Mn nanoferrite, owing to the influence of La^3+^ ions. The photocatalytic mechanism could be explained through the generation of electron–hole pairs when the ferrite surface is exposed to an energy equal to or greater than the band gap energy. Consequently, the photoexcited electron moves from the valence band to the conduction band, creating a hole in the valence band. Effective photocatalysis occurs when these generated pairs remain uncombined. In this scenario, the electrons engage with the adsorbed O_2_ on the photocatalyst’s surface, yielding superoxide radicals, while the holes interact with H_2_O, forming hydroxyl radicals. Both types of radicals are classified as reactive oxygen species capable of breaking down organic pollutant molecules. A significant challenge in this process is the recombination of electron–hole pairs, which hampers the production of reactive oxygen species, thereby impeding the photodegradation process. In our study, the energy levels of dopants, derived from the Ni-ferrite band gap, effectively capture the generated electrons, thereby hindering the recombination of electron–hole pairs. More precisely, part of the photogenerated electrons undergo excitation and reach defect levels, while simultaneously, the photogenerated holes participate in photo-oxidation reactions. The enhanced photocatalytic activity observed in the NiLa_0.3_Fe_1.7_O_4_/SiO_2_ sample can be attributed to its increased adsorption capacity and the introduction of dopant energy levels into the band gap of Ni-ferrite through La substitution. These dopant energy levels serve as mediators for interfacial charge transfer [35], resulting in a high separation rate of photogenerated charge carriers. However, with a higher doping level, La ions become recombination centers, leading to the quenching of photocatalytic activity.

**Figure 9:**
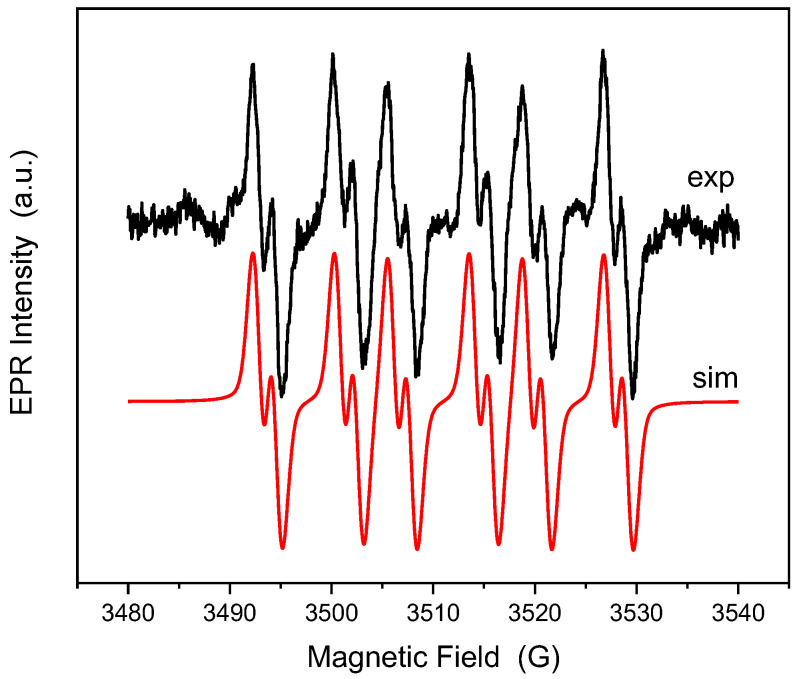
Experimental and simulated spectra of DMPO spin adducts generated by the NiLa_0.3_Fe_1.5_O_4_/SiO_2_ sample after 25 min of irradiation.

To confirm the generation of reactive oxygen species (ROS) by the NiLa_0.3_Fe_1.7_O_4_/SiO_2_ sample under visible irradiation, we utilized EPR spectroscopy coupled with the spin trapping technique. DMPO was employed as the spin trapping agent, and the resulting spectrum is depicted in Figure 9. 

To discern the species accountable for this signal, a simulation was performed. The experimental spectrum fitted closely with the spectrum of the ●DMPO-$O_{2}^{-}$ spin adduct having the following spin Hamiltonian parameters g = 2.0098, ΔH = 1.38 G, a_N_ = 13.2474 G, $a_{H}^{\beta }$ = 8.0109 G, and $a_{H}^{\gamma }$ = 1.6051 G. Unexpectedly, the sample generates only $O_{2}^{-}$, meaning that the maximum valence band position has a lower potential than the oxidation one of the OH-/•OH and H_2_O/•OH redox pair; consequently, these reactions cannot occur [36]. The photocatalytic activity of the sample is exclusively attributed to the generation of superoxide radicals when exposed to visible light. 

The obtained photocatalytic performance results (removal rate and the first-order rate constant, k) for NiLa_0.3_Fe_1.5_O_4_/SiO_2_ annealed at 800 °C are in the same range with other Ni-ferrites previously reported in the literature. Table 3 provides a comparison of various Ni-ferrites, considering both reported work and the current study, with respect to the first-order rate constant.

**Figure 10:**
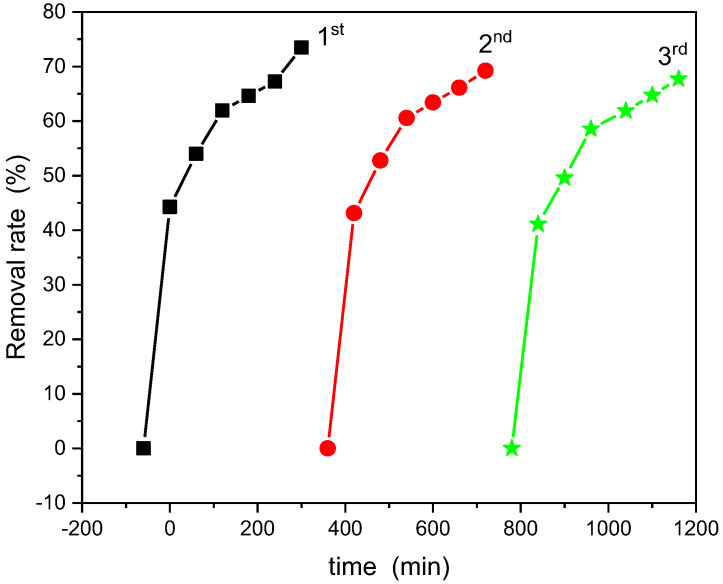
Photocatalyst stability test of NiLa_0.3_Fe_1.7_O_4_/SiO_2_ annealed at 800 °C for removal of RhB.

The photostability of the NiLa_0.3_Fe_1.7_O_4_/SiO_2_ sample (annealed at 800 °C) was verified via reutilization tests in three consecutive trials. The results are depicted in Figure 10. The sample, extracted from the solution using a magnet, underwent a washing with water and ethyl alcohol before each run, followed by an overnight drying. As could be observed from the results, the removal rate shows minimal variation, signifying the robust stability of the photocatalyst.

## 4. Conclusions

Nickel nanoferrite samples doped with La^3+^ ions, featuring various compositions, were synthesized using a sol–gel method. Single-phase nanostructures in the form of an inverse spinel were achieved for Ni-La ferrites across all concentrations at both 500 and 800 °C. The substitution of iron with lanthanum ions within the lattice revealed an expansion of the lattice parameter. This is attributed to the considerable difference in ionic radii between La^3+^ and Fe^3+^, influencing both crystallite size and the fraction of A sites occupied by ferrite cations. Consequently, the degree of the inverse spinel structure experienced an increase. The crystallite size of the mixed Ni-La ferrites increases with the increase in La content and increased temperature, from 19.5 nm to 35.4 nm at 800 °C, from 10.4 nm to 22.6 nm at 500 °C, and from 1.2 nm to 9.8 nm at 200 °C. The particles have an asymmetric spherical shape. The results affirm that the preparation method effectively provided a straightforward means of achieving the desired morphology and microstructure for the ferrite nanocrystals. The specific surface area (SSA) values exhibit variation in accordance with the lanthanum content, showing a decrease as the heat treatment temperature increases. This decline is attributed to the augmentation of grain sizes and crystallinity during the heating process. All nanocomposites present pores in the mesoporous region, with narrow pore size dispersion. All samples show good optical response in the visible range. The best sonophotocatalytic performance was registered for NiLa_0.3_Fe_1.7_O_4_/SiO_2_; this result is most likely because of the La additional levels inserted in the band gap of Ni-ferrite and the equilibrium between La and Fe in Ni-La ferrite.

## Figures and Tables

**Table 1:** Lattice parameter (a), crystallite size (D_C_), particle size from AFM (D_AFM_), H (high), and Rq (roughness) of NiLa_x_Fe_2−x_O_4_/SiO_2_ (x_La_ = 0.1, 0.3, 0.5, 0.7, 0.9, 1.1) annealed at 200, 500, and 800 °C.

NiLa_x_Fe_2−x_O_4_/SiO_2_	Temp, °C	a, Å	D_C_, nm	D_TEM_, nm	D_AFM_, nm	H, nm	Rq, nm	SSA m^2^/g
x_La_ = 0.1	200	-	-	-	10	12	1.23	283
	500	8.444	10.4	-	22	15	1.33	230
	800	8.458	19.5	26	33	11	1.23	3
x_La_ = 0.3	200	8.451	1.2	-	12	14	1.38	267
	500	8.462	12.9	-	25	12	1.17	216
	800	8.481	22.8	31	38	10	1.42	3
x_La_ = 0.5	200	8.469	3.4		15	15	1.43	257
	500	8.483	15.5		28	12	1.15	173
	800	8.497	25.3	35	42	15	2.41	2
x_La_ = 0.7	200	8.488	5.9	-	18	9	0.94	229
	500	8.501	17.8	-	31	13	1.23	208
	800	8.521	28.1	39	44	18	2.77	<0.5
x_La_ = 0.9	200	8.502	8.1	-	20	9	1.01	258
	500	8.517	20.1	-	35	11	1.11	198
	800	8.541	31.7	43	46	22	3.91	<0.5
x_La_ = 1.1	200	8.525	9.8	-	21	12	1.24	-
	500	8.538	22.6	-	37	11	1.14	241
	800	8.566	35.4	47	50	26	4.56	<0.5

**Table 2:** Ni/La/Fe molar ratios of NiLa_x_Fe_2−x_O_4_/SiO_2_ samples calcined at 200, 500, and 800 °C.

x_La_	Ni/La/Fe Molar Ratio			
	Theoretical	200 °C	500 °C	800 °C
0.1	1.0/0.1/1.9	1.01/0.11/1.88	1.00/0.09/1.91	1.00/0.11/1.89
0.3	1.0/0.3/1.7	1.00/0.29/1.71	1.02/0.31/1.67	1.00/0.30/1.70
0.5	1.0/0.5/1.5	1.01/0.52/1.47	1.01/0.49/1.52	1.00/0.49/0.51
0.7	1.0/0.7/1.3	0.99/0.68/1.32	0.98/0.71/1.31	1.01/0.69/1.30
0.9	1.0/0.9/1.1	0.98/0.93/1.09	0.99/0.89/1.12	1.00/0.91/1.09
1.1	1.0/1.1/0.9	1.00/1.11/0.89	1.00/1.08/0.92	1.01/1.09/0.90

**Table 3:** Comparison of various Ni-ferrite samples with respect to the reported first-order rate constant values.

Sample	Lights	Dyes	k × 10^−3^ (min^−1^)	Reference
NiFe_2_O_4_	Visible	Methylene blue	3.4	[4]
NiFe_2_O_4_	Visible	Methyl Orange	2.4	[37]
NiFe_2_O_4_	Visible	Methylene blue	2.3	[38]
NiFe_2_O_4_	Visible	Methylene blue	2.4	[39]
ZnO-NiFe_2_O_4_	Visible	RhB	2.5	[40]
ZnO/NiFe_2_O_4_	Visible	Methylene blue	1.7	[4]
Ni_x_Zn_1−x_Fe_2_O_4_	Sun	Fluorescein	2.7	[41]
Ni_x_Cu_(1−x)_Fe_2_O_4_	Visible	RhB	3.6	[42]
Ni_0.5_Zn_0.5_Fe_2_O_4_	Sun	Methylene blue	6.5	[43]
TiO_2−x_N_x_/SiO_2_/NiFe_2_O_4_	Visible	Methyl Orange	4.7	[44]
NiLa_0.3_Fe_1.5_O_4_/SiO_2_	Visible	RhB	2.3	This work

## Data Availability

Data are contained within the article.
